# Methodological improvements for fluorescence recordings in *Xenopus laevis* oocytes

**DOI:** 10.1085/jgp.201812189

**Published:** 2019-02-04

**Authors:** Elizabeth E.L. Lee, Francisco Bezanilla

**Affiliations:** 1 Committee on Neurobiology, Department of Biochemistry and Molecular Biology, University of Chicago, Chicago, IL; 2 Centro Interdisciplinario de Neurociencia de Valparaiso, Facultad de Ciencias, Universidad de Valparaiso, Valparaiso, Chile

## Abstract

*Xenopus laevis* oocytes are a widely used model system because of their capacity to translate exogenous mRNA, but their high intrinsic background fluorescence is a disadvantage for fluorescence recordings. Here, we developed two distinct methods for improving fluorescence recordings from oocytes. One was a pharmacological method in which a small-molecule salt-inducible kinase inhibitor was co-injected with the mRNA of interest to stimulate melanin production. We interrogated the oocytes using cut-open voltage clamp with simultaneous fluorescence recording and found that by increasing the amount of light-absorbing melanin in these oocytes, we decreased their intrinsic background fluorescence. The treated oocytes produced fluorescence signals that were approximately four times larger. The second method consisted of direct injection of synthetic melanin. This method also significantly improved (doubled) fluorescence signals and allowed any oocyte to be used for fluorescence recording. These two methods provide significant improvements of the signal quality for fluorescent oocyte recordings and allow all healthy oocytes to be used for high-sensitivity recordings.

## Abstract (precis)

*Xenopus laevis* oocytes are used to study membrane proteins because of their ability to translate exogenous mRNA, but their high intrinsic fluorescence limits fluorescence recordings. Lee and Bezanilla present two methods to increase the amount of melanin and reduce background fluorescence in oocytes.

## Introduction

*Xenopus laevis* oocytes are a model in vitro system for development, pharmacology, and electrophysiology (Gurdon et al., 1971). In particular, oocytes have been established as an important system to study membrane proteins and ion channels (Kusano et al., 1977). When voltage-clamp fluorimetry for oocytes was first introduced 20 yr ago (Mannuzzu et al., 1996; Cha and Bezanilla, 1997), it paved the way for novel optical interrogations of membrane proteins with simultaneous electrophysiology. However, optical measurements are currently limited in sensitivity by the oocyte’s high intrinsic background fluorescence in the 300–500 nm wavelength range, thus decreasing the signal-to-noise ratio of recordings in this range.

Recent developments have allowed us to decrease the intrinsic fluorescence of the oocyte through melanin. Melanin is a general name for a group of small light-absorbing polymers. This dark-pigmented molecule plays a role in protection from UV radiation (Costin and Hearing, 2007). Melanin is a useful molecule for suppressing background fluorescence due to its wide range of absorbance (Fig. 4 A). It is well known that the WT expression of melanin in oocytes causes the animal pole to be darker visually and more light absorbent and thus have less fluorescent background compared with the lighter-colored vegetal pole. Thus, we sought to artificially increase the melanin content of the oocyte to further decrease the endogenous fluorescence signal in our recordings.

We demonstrate a novel drug-based method to decrease the endogenous background fluorescence of injected oocytes. A recent study found that the addition of a salt-inducible kinase (SIK) inhibitor increases melanin production in human melanocytes and in vivo in mice (Mujahid et al., 2017). This drug, HG 9-91-01, inhibits SIK2, which in turn stimulates melanogenesis-associated transcription factor, resulting in melanin production by melanosomes. *X. laevis* have homologues for both enzymes and transcription factors, and melanin production can be similarly regulated with HG 9-91-01.

We next demonstrate a second method to decrease endogenous background fluorescence through a direct injection of melanin. For this specific study, we have chosen to use neuromelanin, which is melanin created from the oxidation of dopamine (Zeise et al., 1992). A recent study using synthetic dopamine generated melanin as a photothermal method for treating cancer in vivo (Liu et al., 2013). We used this method of synthesizing melanin, but with the aim of reducing the fluorescence background to improve oocyte recordings. These methodological improvements offer many opportunities for better fluorescence recordings in oocytes.

## Materials and methods

### Oocyte preparation

Oocytes were harvested from *X. laevis* in accordance with experimental protocols approved by the University of Chicago Animal Care and Use Committee. Following collagenase digestion of the follicular membrane, oocytes were stored at 18°C in standard oocyte solution containing (in mM) 96 NaCl, 2 KCl, 1.8 CaCl_2_, 1 MgCl_2_, and 10 HEPES, pH 7.4, with 10 mg/liter gentamicin. Oocytes were typically injected 1–2 d after digestion.

### Molecular biology

ASAP1 for *X. laevis* expression was created from the pcDNA3.1/Puro-CAG-ASAP1, which was a gift from Michael Lin (Stanford University, Stanford, California; plasmid 52519; Addgene; St-Pierre et al., 2014). ASAP-Y was identical to ASAP1 with an additional point mutation at L158Y (Lee and Bezanilla, 2017). Using site-directed mutagenesis by PCR, we added L158Y to this construct and verified it via sequencing.

Shaker complementary RNA (cRNA) was used that had been previously synthesized using the mMESSAGE mMACHINE T7 transcription kit (Life Technologies). Shaker W434F M356C was made on the Shaker Δ6-46 W434F background (Perozo et al., 1993) using the QuikChange II site directed mutagenesis kit (Agilent) with primers purchased from Integrated DNA Technologies. DNA was linearized using *Not*I (New England Biolabs).

For oocyte expression, DNA was prepared using the NucleoSpin Plasmid kit (Macherey-Nagel) and linearized with *Xba*I (New England Biolabs). Linearized cDNA was transcribed to RNA with the mMESSAGE mMACHINE Sp6 kit (Life Technologies). Oocytes were injected with 50 ng RNA and incubated at 18°C in solution containing (in mM) 96 NaCl, 2 KCl, 1.8 CaCl_2_, 1 MgCl_2_, and 10 HEPES, pH 7.4, with 10 mg/liter gentamicin. Recordings were made 1–2 d following injection.

### Electrophysiology and fluorescence

Simultaneous recordings of gating currents and fluorescence responses were performed using the cut-open oocyte voltage-clamp technique (Stefani and Bezanilla, 1998) in combination with a photodiode to measure temporal changes in fluorescence emission (Cha and Bezanilla, 1998). Gpatch, an in-house program, controlled an SB6711 digital signal processor-based board (Innovative Integration) with an A4D4 board (Innovative Integration). Oocytes were held under voltage clamp with a Dagan CA-1B amplifier and current was filtered at 5 kHz. ASAP-Y was sampled at 50 kHz and fluorescence emissions were collected through an Olympus LUMPlan FL N ×40/0.8 numerical aperture water-immersion objective by a PIN-020A photodiode (UDT Technologies), amplified by a patch-clamp amplifier L/M-EPC-7 by LIST Medical Electronic with a filter of 10 kHz, and then integrated over each sampling period using a home-built integrator circuit. ASAP-Y was excited via a ThorLabs LED controller powering a 470-nm LED (ThorLabs) that was passed through a filter cube housing a 480/40-nm excitation filter, a 505-nm long-pass dichroic, and a 535/50-nm emission filter (Chroma). For oocytes stained with ATTO-425, excitation was performed with a mounted 420-nm LED (ThorLabs) reflected by a 455-nm long-pass dichroic (Chroma) through a ×40 water-immersion objective (LUMPlan FL N; Olympus); emission was collected through the dichroic and a 475-nm long-pass filter (Chroma). All recordings were performed at ∼18°C, with an external solution containing (in mM) 115 N-methyl-d-glucamine neutralized with methanesulfonic acid, 10 HEPES, and 2 Ca(OH)_2_, and an internal solution containing (in mM) 115 N-methyl-d-glucamine neutralized with methanesulfonic acid, 10 HEPES, and 2 EGTA. Both solutions were set to pH 7.5. Microelectrodes were pulled on a Flaming–Brown micropipette puller (model P-87; Sutter Instruments) and were filled with 3 M CsCl_2_ and had a resistance of ∼0.2–0.8 MΩ.The labeling solution consisted of depolarizing solution comprised of 120 mM KCl, 2 mM CaCl_2_, and 10 mM HEPES, pH 7.4, with 20 µM ATTO 425 maleimide (ATTO-TEC). Oocytes were maintained in the solution on ice for at least 15 min and then removed and washed in standard oocyte solution 5–20 min before recordings were performed.

### Chemical biology

HG 9-91-01 (MedChem Express) was dissolved in DMSO to create a stock solution of 200 mM. Then, with 200-µM aliquots diluted with RNase-free water (Ambion), HG 9-91-01 was injected at concentrations stated in the paper in Figs. 2 and 3. When used with cRNA, HG 9-91-01 was mixed and coinjected. For example, the 200 µM HG 9-91-01 was mixed 1:1 with 2,000 ng/μl cRNA of nonconductive Shaker constructs for injecting 100 µM. DMSO concentration, when injected into the oocyte, was less than 0.1%. Since the volume of an oocyte is ∼1 μl and we injected 50.1 nl, the final concentration of HG 9-91-01 inside the oocyte was ∼4.7 µM.

For synthesis of synthetic melanin with an average diameter of 200 nm, 3 ml ammonia aqueous solution (NH_4_OH, 28–30%; Sigma) was mixed with 40 ml of 200-proof ethanol (Decon Laboratories) and 90 ml Milli-Q water (Millipore) with mild stirring at 30°C for 30 min. 0.5 g dopamine hydrochloride (Sigma) was dissolved in 10 ml Milli-Q water and injected into the above solution. The color of the solution immediately turns pale yellow and quickly darkens to brown. The reaction was left at room temperature for 24 h. The melanin mixture was centrifuged and rinsed with copious amounts of Milli-Q water. To create different concentrations, the melanin was dried, and weighed, and resuspended in RNase-free water (Ambion) before injection.

### Melanin characterization

Absorbance was measured on a Cary 60 UV-Vis (Agilent Technologies).

Dynamic light scattering was done on a Dynapro Nanostar (Wyatt Technology) of an unfiltered 30-mg/ml sample of synthetic melanin. We took 10-μl aliquots of synthetic melanin and measured in 10 5-s acquisitions. We used the globular proteins model for calculating the radius with an Rg Model of Sphere.

### Statistical analysis

**Figure 1.**
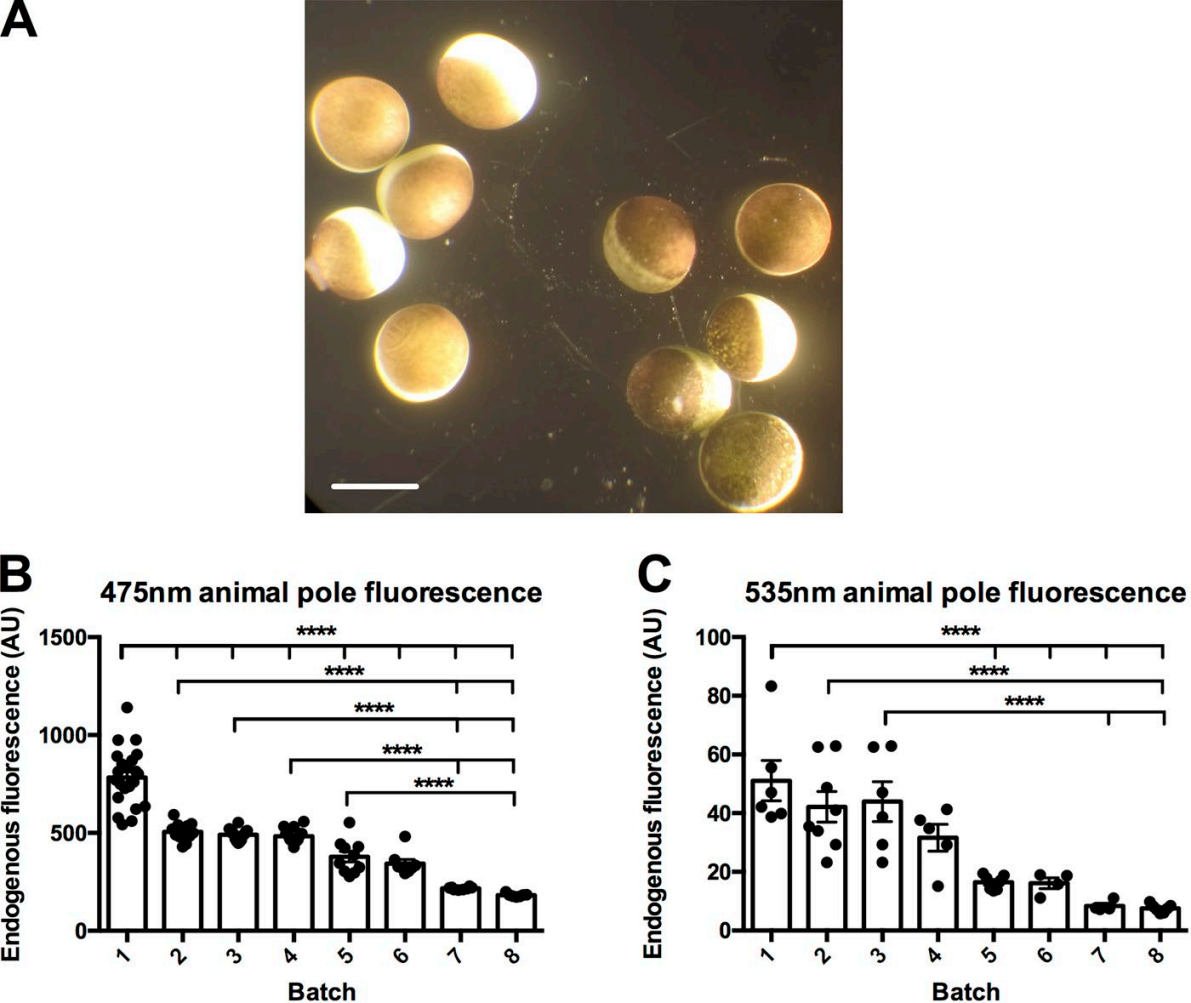
**Oocytes are variable in endogenous background fluorescence at multiple wavelengths. (A)** *X. laevis* oocytes from different batches of oocytes. Scale bar, 1 mm. **(B)** Endogenous background fluorescence of the animal pole excited with a 420-nm LED reflected onto the oocytes by a 455-nm long-pass dichroic through a ×40 water-immersion objective, where the emission was collected through the dichroic and a 475-nm long-pass filter. Batches were placed in order of most endogenous background to least. Batch 1, *n* = 22; batch 2, *n* = 1; batch 3, *n* = 14; batch 4, *n* = 20; batch 5, *n* = 10; batch 6, *n* = 8; batch 7, *n* = 8; and batch 8, *n* = 7. A one-way ANOVA together with a post hoc Tukey multiple comparisons test. ****, P < 0.0001. For further information, see Table S1. **(C)** Endogenous background fluorescence of the animal pole excited with a 470-nm LED that was passed through a filter cube housing a 480/40-nm excitation filter, a 505-nm long-pass dichroic, and a 535/50-nm emission filter. Batches are placed in order of most endogenous background to least. Batch 1, *n* = 6; batch 2, *n* = 8; batch 3, *n* = 6; batch 4, *n* = 5; batch 5, *n* = 7; batch 6, *n* = 4; batch 7, *n* = 4; and batch 8, *n* = 7. A one-way ANOVA together with a post hoc Tukey multiple comparisons test. ****, P < 0.0001. For further information, see Table S2. The batch numbers in B and C do not correspond and are different batches of oocytes. The measurements are in comparable optical conditions.

**Figure 2.**
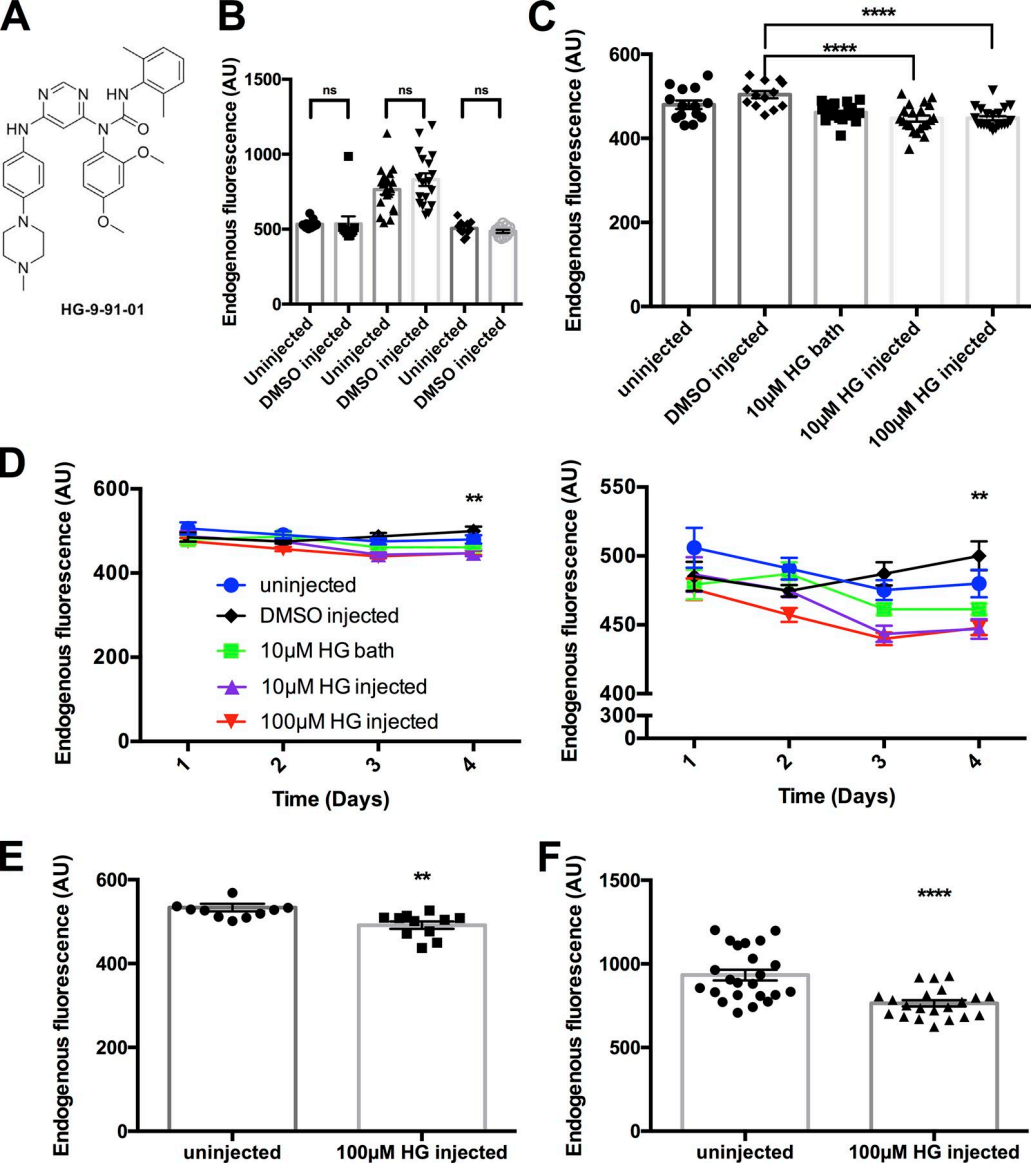
**Addition of the SIK inhibitor decreases background fluorescence. (A)** Chemical structure of the small-molecule SIK inhibitor HG 9-91-01. **(B)** Comparison of endogenous fluorescence between several batches of uninjected and 0.5% DMSO-injected oocytes. A one-way ANOVA with a post hoc Holm–Sidak multiple comparisons test found no significant difference compared with uninjected oocytes (uninjected: *n* = 11, 19, and 11; DMSO: *n* = 10, 18, and 11). For further information, see Table S3. **(C)** Effect of HG 9-91-01 endogenous fluorescence under different conditions: uninjected (circles, *n* = 14), 0.1% DMSO injected (diamonds, *n* = 9), 10 µM HG 9-91-01 bath (squares, *n* = 22), 10 µM HG 9-91-01 injected (triangles, *n* = 21), and 100 µM HG 9-91-01 injected (upside-down triangles, *n* = 21) measured on d 4. ****, P < 0.0001 compared to uninjected oocytes, one-way ANOVA with a post hoc Holm–Sidak multiple comparisons test. For further information, see Table S4. **(D)** Endogenous fluorescence under different conditions measured over time, as described in B. Panel on the right is a zoom in to demonstrate the effect of the different conditions described in B. **, P < 0.001, 10 µM HG injected and 100 µM HG injected on day 4 (two-way ANOVA with a post hoc Dunnett multiple comparisons test). For further information, see Table S5. **(E)** Results from a darker batch of oocytes where HG 9-91-01 was tested (uninjected: *n* = 11; 100 µM HG 9-91-01 injected: *n* = 11). **, P = 0.0031, unpaired two-tailed *t* test. **(F)** Results from a lighter batch of oocytes where HG 9-91-01 was tested (uninjected: *n* = 22; 100 µM HG 9-91-01 injected: *n* = 14). ****, P < 0.0001, unpaired two-tailed *t* test. All error bars are ±SEM centered on the mean..

**Figure 3.**
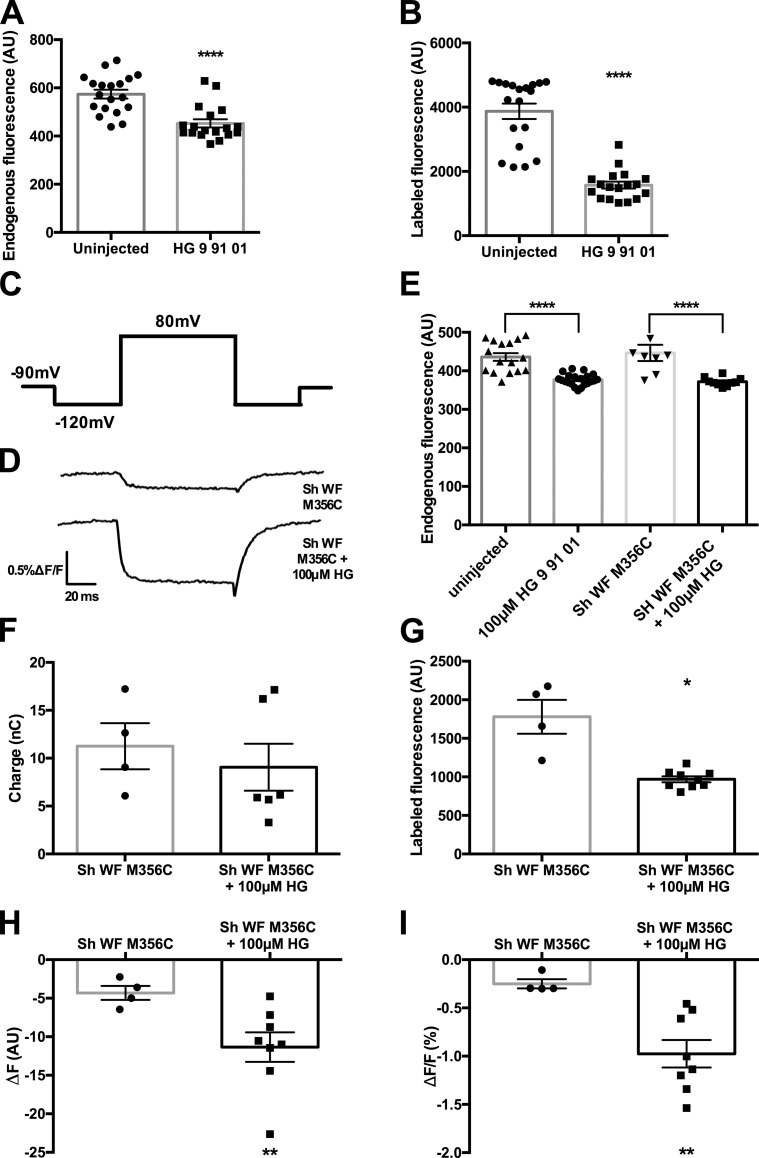
**Addition of the SIK inhibitor improves ATTO 425 signal. (A)** A comparison of uninjected (*n* = 19) and 100 µM HG 9-91-01–injected (*n* = 18) oocytes on day 4 before labeling with ATTO 425. **(B)** ****, P < 0.0001 after labeling with ATTO 425, unpaired two-tailed *t* tests. **(C)** Pulse protocol for ATTO 425 fluorescence, where the oocytes were held at −90 mV and prepulsed to −120 mV for 60 ms, followed by an 80-ms pulse to 80 mV, with a post pulse of 120 mV for 60 ms before returning to the holding potential. **(D)** Representative trace of fluorescence data of ATTO 425–labeled Shaker W434F M356C (top) and ATTO 425 labeled Shaker W434F M356C + 100 µM HG 9-91-01 (bottom) oocytes with the pulse protocol from A. **(E)** Endogenous oocyte fluorescence on day 3 was checked for several conditions (uninjected, *n* = 16; construct, *n* = 8; HG 9-91-01, *n* = 21; construct + HG 9-91-01, *n* = 10). ****, P < 0.0001, ordinary one-way ANOVA with a post hoc Bonferroni multiple comparisons test. **(F)** Comparisons of levels of expressions in Shaker W434F M356C (*n* = 4) and Shaker W434F M356C + HG 9-91-01 (*n* = 6) injected oocytes. Unpaired two-tailed *t* test showed no statistical significance (P = 0.5414). **(G)** Comparison of background fluorescence of ATTO 425–labeled oocytes Shaker W434F M356C (*n* = 4) and Shaker W434F M356C + HG 9-91-01 (*n* = 8). *, P = 0.0324, unpaired two-tailed *t* test. **(H)** Comparisons of fluorescence signal in oocytes injected with Shaker W434F M356C (*n* = 4) and Shaker W434F M356C + HG 9-91-01 (*n* = 8). **, P = 0.0085, unpaired two-tailed *t* test. **(I)** Comparison of ΔF/F % in oocytes injected with Shaker W434F M356C (*n* = 4) and Shaker W434F M356C + HG 9-91-01 (*n* = 8). **, P = 0.0011, unpaired two-tailed *t* test. For further information, see Tables S6–S12. All error bars are ±SEM centered on the mean.

Tables S1–S19 show the results of the indicated statistical tests performed on data presented in Figs. 1, B and C; 2, B–D; 3, A, B, and E–I; 4 B, 5, C and D; and 6, C–F. Tables S1 and S2 show the statistical significance of oocyte endogenous fluorescence by batch variance, as established by ordinary one-way ANOVA with a post hoc Tukey multiple comparisons test. To establish a lack of statistical significance between uninjected and DMSO-injected oocytes, we used an ordinary one-way ANOVA with a post hoc Holm–Sidak multiple comparison test. Table S3 shows that within batches, there was no significant difference. To establish the statistical significance of the effect of HG 9-91-01 on background fluorescence of the oocyte, we used an ordinary one-way ANOVA with a post hoc Holm–Sidak (Table S4), Dunnett (Table S5), and Bonferroni (Table S6) multiple comparison test. To establish the effect of HG 9-91-01 on the labeling of oocytes, we first showed that HG 9-91-01 significantly decreased the endogenous fluorescence using an unpaired two-tailed *t* test (Table S7). Then, also using an unpaired two-tailed *t* test, we showed the difference after ATTO 425 labeling in uninjected and HG 9-91-01 injected oocytes (Table S8). Tables S9–S12 show the statistical significance of the effect of HG 9-91-01 on gating charge (Table S9), labeled background fluorescence (Table S10), ΔF (Table S11), and ΔF/F% (Table S12) as established using an unpaired two-tailed *t* test. Table S13 shows the statistical significance of the effect of melanin injection on background fluorescence as established by an ordinary two-way ANOVA with a post hoc Tukey multiple comparison test in several batches. Tables S14 and S15 show the statistical significance of the effect of melanin injections on background fluorescence as established by an ordinary one-way ANOVA with a post hoc Tukey multiple comparison test for batches of darker oocytes (Table S14) and lighter oocytes (Table S15). Table S16 shows the statistical insignificance of melanin injection on the amount of gating charge as demonstrated by an unpaired two-tailed *t* test. Tables S17 to S19 show the statistical significance of the effect of melanin injection on F (Table S17), ΔF (Table S18), and ΔF/F% (Table S19) of ASAP-Y was established with an ordinary one-way ANOVA with a post hoc Tukey multiple comparison test. 

### Data analysis and figures

Data analysis and figures were made with Prism 6.0 (GraphPad Software) and Analysis (in-house program).

### Online supplemental material

**Figure 4.**
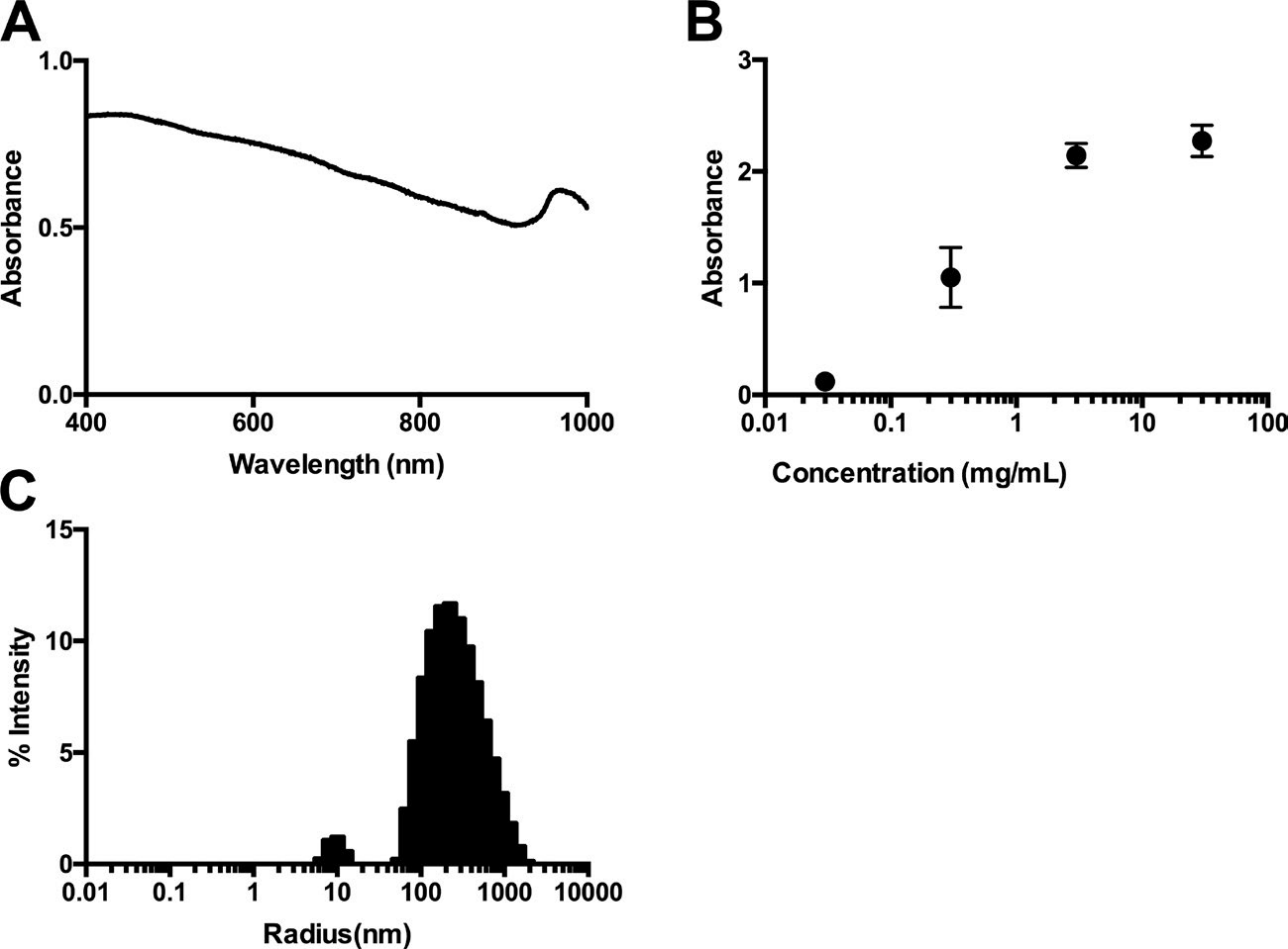
**Synthetic melanin characterization. (A)** Absorption spectrum of 0.3 mg/ml melanin from 400 to 1,000 nm. **(B)** Absorbance of different concentrations of melanin at 600 nm (*n* = 3). **(C)** Dynamic light scattering measurement of synthetic melanin size at 30 mg/ml unfiltered (average radius size, 220 nm).

**Figure 5.**
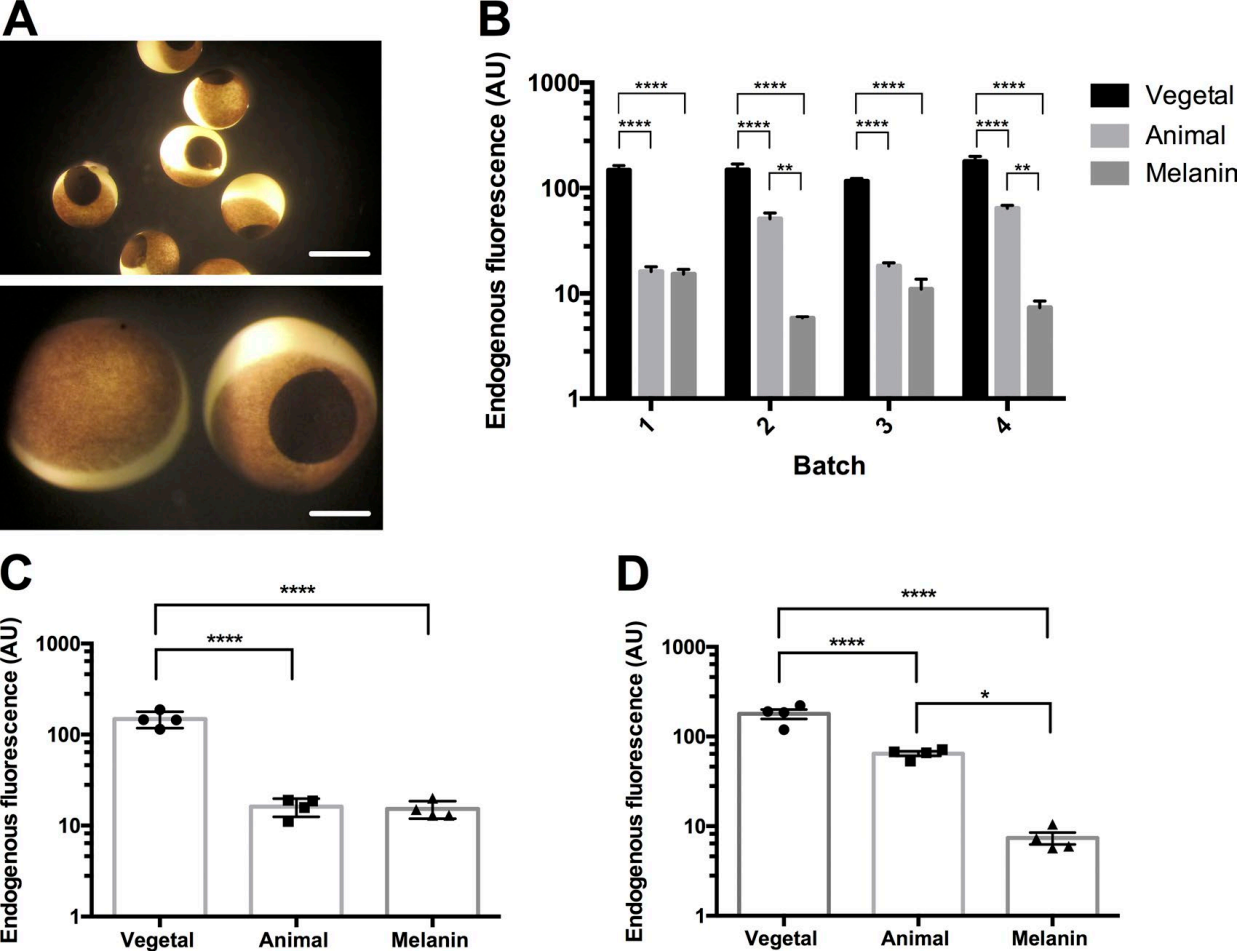
**Synthetic melanin injections significantly improve fluorescence recording conditions. (A)** Representative *X. laevis* oocytes injected with 50.1 nl of 30 mg/ml melanin. Scale bars, 1 mm (top) and 0.3 mm (bottom). **(B)** Improvement of endogenous background fluorescence of four separate batches of oocytes at 535 nm. Batch 1, *n* = 4; batch 2, *n* = 6; batch , *n* = 10; and batch 4, *n* = 4. *, P < 0.05; **, P < 0.01; ****, P < 0.0001, two-way ANOVA with a post hoc Tukey multiple comparison test. **(C)** The improvement in endogenous background fluorescence in “dark” oocytes, <20 AU at 535 nm (vegetal, *n* = 4; animal, *n* = 4; and melanin injected, *n* = 4). ****, P < 0.0001, ordinary one-way ANOVA with a post hoc Tukey multiple comparison test; no statistically significant difference was found between the animal pole and melanin. **(D)** The improvement in endogenous background fluorescence in “light” oocytes, >20 AU at 535 nm (vegetal, *n* = 4; animal, *n* = 4; and melanin injected, *n* = 4). *, P < 0.05; ****, P < 0.0001, ordinary one-way ANOVA with a post hoc Tukey multiple comparison test. For further statistical information, see Tables S13–S15.

**Figure 6.**
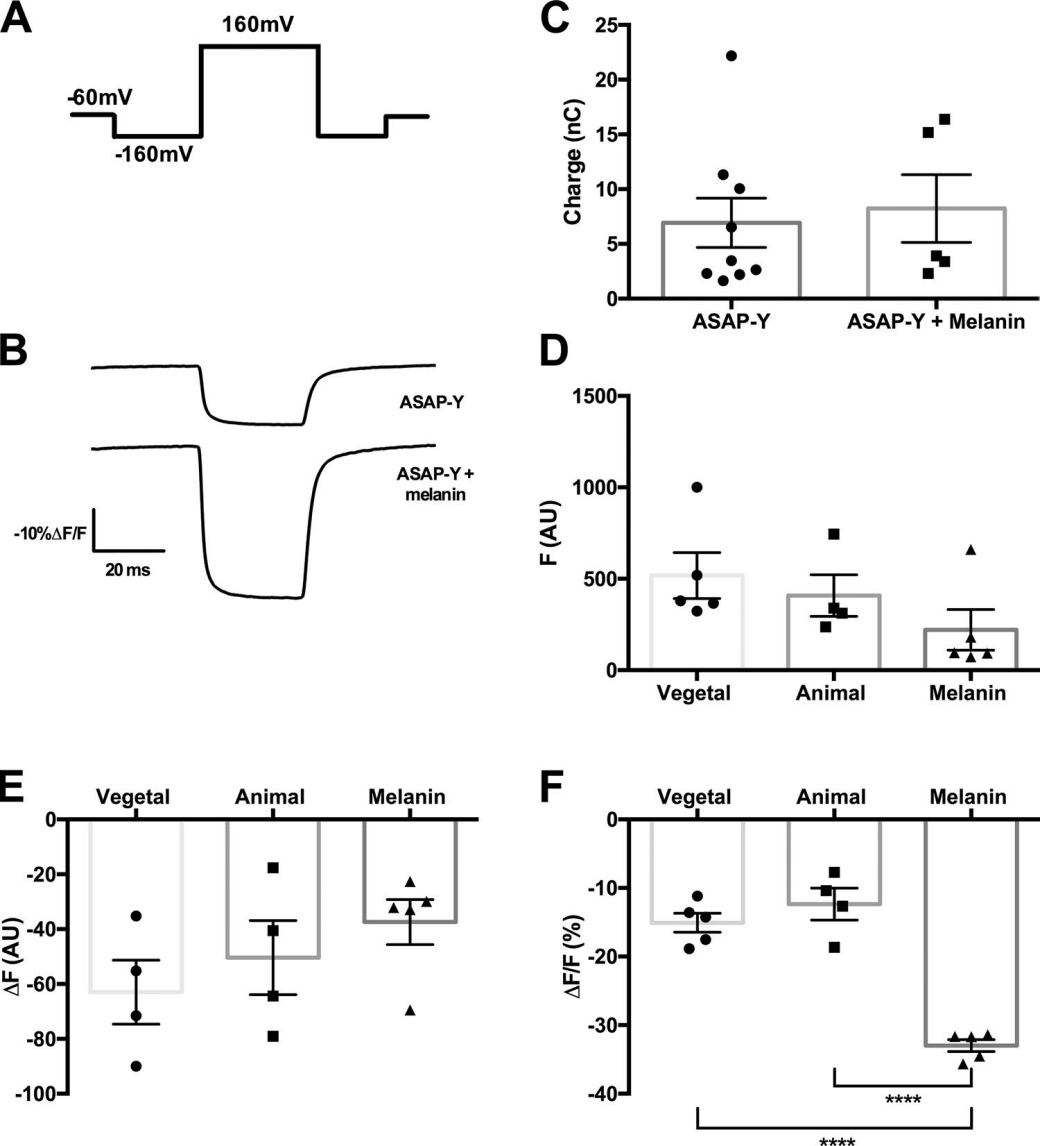
**Synthetic melanin injections improve the signal of ASAP-Y. (A)** Pulse protocol for ASAP-Y fluorescence, where the oocytes were held at −60 mV with a 50-ms prepulse to −160 mV, a 30-ms pulse to 160 mV, and a postpulse back to −160 mV for 50 ms before returning to holding. **(B)** A representative trace of fluorescence data of ASAP-Y (top) and ASAP-Y and melanin (bottom) with the pulse protocol from A. **(C)** Comparisons of levels of expressions in oocytes injected with ASAP-Y (*n* = 9) and ASAP-Y and melanin (*n* = 5). P = 0.7365, unpaired two-tailed *t* test showed no statistical significance. **(D)** Comparison of background fluorescence at a holding potential of −60 mV in ASAP-Y (vegetal, *n* = 5; animal, *n* = 4) and ASAP-Y and melanin (*n* = 5). An ordinary one-way ANOVA with a post hoc Tukey multiple comparison test found no statistically significance differences among the three conditions. **(E)** Comparisons of fluorescence signal in oocytes injected with ASAP-Y (vegetal, *n* = 5; animal, *n* = 8) and ASAP-Y and melanin (*n* = 5). An ordinary one-way ANOVA with a post hoc Tukey multiple comparison test found no statistically significance differences among the three conditions. **(F)** Comparisons of ASAP-Y–injected oocytes ΔF/F % (vegetal, *n* = 5; animal, *n* = 4; and melanin injected, *n* = 5). ****, P < 0.0001, ordinary one-way ANOVA with a post hoc Tukey multiple comparison test. For further statistical information, see Tables S16–S19. All error bars are ±SEM centered on the mean.

The supplemental statistical tables detail the statistical tests performed for each of the comparisons in Figs. 1, 2, 3, 4, 5, and 6 as described above in the statisical analysis section. 

## Results

### Oocytes are variable in endogenous background fluorescence at different wavelengths

We first sought to quantify the endogenous fluorescence of oocytes before RNA injection (uninjected oocytes) at two different wavelengths of excitation and emission. We excited oocytes with 420-nm and 470-nm LEDs and measured emissions from a 475-nm long-pass and a 535/50-nm emission filter, respectively (Fig. 1, B and C). The endogenous fluorescence of oocytes varies by batch and wavelength (Fig. 1). This variability can limit opportunities to do fluorescence recordings if the oocytes have an intrinsically light animal pole (high fluorescence background) color. In the extreme case, the variability may exclude some batches of oocytes from use for fluorescence recordings. We thus aimed to develop several methods to attenuate the fluorescence variability of oocytes.

### Addition of injected HG 9-91-01 decreased endogenous background oocyte fluorescence

We recorded fluorescence from the animal pole of the *X. laevis* oocyte, exciting at 420 nm and collecting the emission with a 475-nm long-pass filter. A genetic sequence analysis revealed homologues to both SIK2 and melanogenesis-associated transcription factor in *X. laevis*, both of which are in the pathway affected by HG 9-91-01 (Fig. 2 A; Mujahid et al., 2017). To ensure that the DMSO in which HG 9-91-01 was solubilized was not having an effect, we checked several batches and found no difference between these two groups (Fig. 2 B). Because in the original study the inhibitor was topically applied, we first attempted bath application of this drug, which did not improve oocyte endogenous fluorescence (Fig. 2, C and D). We then performed a dose–response injection of the drug into the oocyte and found that 100 µM HG 9-91-01 decreased the endogenous oocyte fluorescence (Fig. 2, C and D). Interestingly, there was a limit to how much the signal could be improved. In intrinsically dark oocytes (200–400 AU of fluorescence at 475 nm), we did not observe as large a change in endogenous fluorescence (Fig. 2 E); however, in “lighter” oocytes (>500 AU of fluorescence at 475 nm), there is an improvement (Fig. 2 F).

To determine if this method would improve the signal-to-noise ratio of fluorescence recordings in an experimental setting, we used dye-labeled nonconductive Shaker mutants. We measured the fluorescence signal of ATTO 425 (Kubota et al., 2014); when oocytes are labeled with this dye, it causes an increase in the measured background fluorescence. We first looked at the effects of dye labeling on uninjected and 100 µM HG 9-91-01–injected oocytes (Fig. 3 A) and saw that labeling with ATTO 425 greatly increased the background-labeled fluorescence of both groups of oocytes (Fig. 3 B). This increase in fluorescence could be caused by partitioning of dye into the membrane or even penetration of some dye into the oocyte itself (Toseland, 2013; Hughes et al., 2014). The important result here is that after dye labeling, we observed a lower intensity of fluorescence in HG 9-91-01–injected oocytes due to the increased internal melanin, which blocks the signal of internalized dye molecules, in comparison to uninjected dye-labeled oocytes (Fig. 3 B).

We conjugated this dye via cysteine to a mutant of a nonconductive Shaker mutant M356C in the S3-S4 linker region (Cha and Bezanilla, 1997). This dye gave a robust voltage-dependent signal at this location (Fig. 3, C and D). We chose a batch of lighter oocytes to demonstrate the effect of HG 9-91-01 (Fig. 3 E). The co-injection of HG 9-91-01 did not alter the expression of the Shaker construct, as we compared total gating charge in both conditions as a measure of expression (Fig. 3 F). We did not observe an alteration in the currents recorded with HG 9-91-01 co-injection.

Following a labeling step with ATTO 425 and a wash, we observed a much lower intensity of fluorescence from oocytes coinjected with HG 9-91-01, despite a comparable amount of charge measured with Sh WF M356C–injected oocytes (Fig. 3 G). With the labeling, the large labeled background fluorescence masks the size of the voltage-induced fluorescence signal even when expression is high.

To investigate whether the voltage-dependent signal was improved, the oocytes were depolarized to 80 mV for 80 ms. Based on previous experiments, the largest signal is generated at 80 mV and could best demonstrate the effect of HG 9-91-01 on signal size (Fig. 3 H). There is a ∼0.25% ΔF/F in oocytes injected with Sh WF M356C only; however, in HG 9-91-01–treated oocytes, we saw ∼1% ΔF/F, a fourfold increase in ΔF/F (Fig. 3 I). Thus, the voltage-dependent fluorescence signal was larger, mainly because of the decrease in background fluorescence by the effect of SIK inhibitor.

### Synthetic melanin injections significantly improve the signal-to-noise ratio

The reduced endogenous fluorescence by HG 9-91-01 treatment to increase endogenous production of melanin led us to ask if a direct injection of synthetic melanin could improve the signal-to-noise ratio even more dramatically. While Liu et al. (2013) focused on the photothermal effects of melanin for cancer treatments, we used their technique of synthesizing melanin and applied it as a methodological improvement of endogenous oocyte fluorescence. Melanin is a useful molecule due to its wide range of absorbance as well as its linear relationship to log of the concentration (Fig. 4, A and B). Using their method, we synthesized two sizes of melanin averaging 220 nm and 500 nm, as determined by dynamic light scattering (Fig. 4 C). We used the 220-nm melanin particles because, at larger sizes, injection is not possible, as the injection pipette clogs.

We injected each oocyte with 30 mg/ml melanin in 50.1 nl per oocyte in various regions. The injection of melanin is done superficially, creating a disc of melanin ∼0.5 mm in diameter near the surface of the oocyte (Fig. 5 A, bottom). Injecting melanin at either the vegetal or animal pole reduced the endogenous fluorescence. For the remaining experiments, we injected the melanin specifically on the animal pole (Fig. 5 B). We found that, depending on the intrinsic absorbance of oocytes, injecting the melanin into the animal pole decreased the endogenous fluorescence. In the darker batches of oocytes, the melanin disc had comparable or slightly decreased fluorescence with reference to the fluorescence of the animal pole (Fig. 5 C). In lighter batches (Fig. 5 D), the fluorescence of the melanin was significantly lower than that of the animal pole. Therefore, across all batches of oocytes, we can inject synthetic melanin to make fluorescence recordings feasible. To see how this method affects the oocytes in an experimental scheme, we co-injected the melanin in a lighter batch of oocytes (Fig. 5 D) with mRNA for ASAP-Y (Lee and Bezanilla, 2017), a genetically encoded voltage indicator (Fig. 6). We chose to attempt these experiments in lighter oocytes to see how well the melanin would improve the signal. To investigate whether the voltage-dependent signal was improved, the oocytes were depolarized to 160 mV for 80 ms (Fig. 6 A). Based on previous experiments, the largest signal was generated at 160 mV and best demonstrated the effect of melanin on signal size (Fig. 6 B).

The melanin did not alter the expression of the genetically encoded voltage indicator, as we saw no change in the amount of charge or statistically significant change in the amount of fluorescence when at a holding potential of −60 mV (Fig. 6, C and D), but we see a large optical improvement of approximately twice as large for the ΔF/F (Fig. 6, E and F). This indicates that by decreasing the endogenous fluorescence, we were able to achieve an overall larger ASAP-Y signal.

## Discussion

The melanin layer located under the plasma membrane acts as barrier for the endogenous fluorescence when doing fluorescence recordings from the animal pole in *Xenopus* oocytes. This endogenous fluorescence varies by batch due to variability in the intrinsic production of melanin (Fig. 1). This variability can limit opportunities to perform fluorescence recordings if the oocytes have too high an endogenous background fluorescence. The studies presented here introduce two different methods to improve fluorescence recording conditions. One method increases production of the endogenous melanin, whereas the second method injects synthetic melanin directly into the oocyte. Both methods significantly decrease in the background fluorescence and thus improve conditions to acquire fluorescence recordings in batches of lighter oocytes. We have also found that this extra melanin helps in reducing the increased background fluorescence resulting from staining an oocyte with a fluorophore intended to be conjugated to a particular site in an expressed membrane protein. This effect is shown when we stained the oocyte with ATTO 425 and found that the increase in background fluorescence induced by the dye was 3.8 times larger than the intrinsic fluorescence, while, in contrast, the observed increase after HG 9-91-01 injection was only 2.8 times larger (Fig. 3, E and G). Many dyes partition in the membrane and remain there even after extensive washes, and this is the case for ATTO 425. In principle, if the dye is only in the membrane, then one would not expect that an increase in melanin would be advantageous. However, there are at least three possible scenarios where the increase in melanin would help in decreasing the extra fluorescence of stained oocytes. First, some dye may penetrate; second, there might be energy transfer from the membrane to the endogenous fluorophores; and third, the extra fluorescence from the dye in the membrane may be reflected (scattered) by the cytoplasm.

As the treatments presented here do not affect the expression of the protein under study, the improved recording conditions allow for significant increases in the efficacy and efficiency of oocyte experiments. Future studies may be needed to see possible effects of either HG 9-91-01 or melanin on internal second messengers such as (but not limited to) calcium or cAMP. These treatments eliminate the need to discard batches of oocytes if they have high endogenous fluorescence. Furthermore, it demonstrates the feasibility of improving the signal-to-noise ratio in fluorescence recordings by a pharmacologic method and argues for the investigation of other pharmacologic methods with the potential to lower endogenous background. These methods could allow for the resolving of signals that were too small to be previously seen as well as permit an exploration of kinetics. The method of injecting melanin could potentially also improve the study of proteins that preferentially express in the vegetal pole, such as GIRK5 and IP_3_R in unfertilized oocytes (Kume et al., 1993; Díaz-Bello et al., 2013; Sindelka et al., 2018). Moreover, it is possible for this method to be applied to other experimental systems where endogenous fluorescence is a systematic problem, such as total internal reflection microscopy, single-molecule tracking, and fluorescence resonance energy transfer studies.

## Supplementary Material

Supplemental Materials (PDF)

Tables S1-S19 (PDF)
