# Supplementary material for: Methodological improvements for fluorescence recordings in Xenopus laevis oocytes
Source: J Gen Physiol. 2019 Feb 4;151(2):264–72. doi: 10.1085/jgp.201812189 (PMC6363413; doi:10.1085/jgp.201812189)
Supplement: Supplemental Materials (PDF) [file JGP_201812189_sm.pdf]

Supplemental material

Lee and Bezanilla, https://doi.org/10.1085/jgp.201812189

Supplemental tables are provided as a separate PDF file.
