# Supplementary material for: Methodological improvements for fluorescence recordings in Xenopus laevis oocytes
Source: J Gen Physiol. 2019 Feb 4;151(2):264–72. doi: 10.1085/jgp.201812189 (PMC6363413; doi:10.1085/jgp.201812189)
Supplement: Tables S1-S19 (PDF) [file JGP_201812189_TableS1.pdf]

Table S1. Fig. 1 B

One-way ANOVA	
DoF	7.92
F	75.36
P	<0.0001
Statistical significance	****

Tukey multiple comparison test	Mean difference	95% CI of difference	Summary
1 vs. 2	8.946	−10.33 to 28.22	NS
1 vs. 3	7.143	−13.47 to 27.75	NS
1 vs. 4	19.46	−2.151 to 41.08	NS
1 vs. 5	34.65	14.80 to 54.51	****
1 vs. 6	34.93	11.89 to 57.97	***
1 vs. 7	42.71	19.67 to 65.76	****
1 vs. 8	43.52	23.66 to 63.38	****
2 vs. 3	−1.803	−21.08 to 17.47	NS
2 vs. 4	10.52	−9.832 to 30.87	NS
2 vs. 5	25.71	7.234 to 44.18	**
2 vs. 6	25.98	4.125 to 47.84	*
2 vs. 7	33.77	11.91 to 55.63	***
2 vs. 8	34.57	16.10 to 53.05	****
3 vs. 4	12.32	−9.294 to 33.94	NS
3 vs. 5	27.51	7.652 to 47.37	**
3 vs. 6	27.79	4.745 to 50.83	**
3 vs. 7	35.57	12.53 to 58.61	***
3 vs. 8	36.37	16.52 to 56.23	****
4 vs. 5	15.19	−5.711 to 36.09	NS
4 vs. 6	15.47	−8.479 to 39.41	NS
4 vs. 7	23.25	−0.6952 to 47.20	NS
4 vs. 8	24.05	3.153 to 44.96	*
5 vs. 6	0.2757	−22.10 to 22.65	NS
5 vs. 7	8.06	−14.31 to 30.43	NS
5 vs. 8	8.864	−10.22 to 27.94	NS
6 vs. 7	7.784	−17.46 to 33.02	NS
6 vs. 8	8.588	−13.79 to 30.96	NS
7 vs. 8	0.8041	−21.57 to 23.18	NS

CI, confidence interval; DoF, degrees of freedom. *, P < 0.05; **, P < 0.01; ***, P < 0.001; ****, P < 0.0001.

Table S2. Fig. 1 C

One-way ANOVA	
DoF	7,39
F	14.18
P	< 0.0001
Statistical significance	****

Tukey multiple comparison test	Mean difference	95% CI of difference	Summary
1 vs. 2	277.2	184.5 to 369.8	****
1 vs. 3	292.3	206.6 to 378.1	****
1 vs. 4	299.5	222.0 to 377.0	****
1 vs. 5	404.8	309.1 to 500.5	****
1 vs. 6	439.9	336.3 to 543.4	****
1 vs. 7	566.5	462.9 to 670.1	****
1 vs. 8	601	492.1 to 709.9	****
2 vs. 3	15.17	−85.92 to 116.3	NS
2 vs. 4	22.3	−71.88 to 116.5	NS
2 vs. 5	127.6	17.95 to 237.2	*
2 vs. 6	162.7	46.09 to 279.3	***
2 vs. 7	289.3	172.7 to 405.9	****
2 vs. 8	323.8	202.5 to 445.1	****
3 vs. 4	7.131	−80.30 to 94.56	NS
3 vs. 5	112.4	8.526 to 216.3	*
3 vs. 6	147.5	36.30 to 258.7	**
3 vs. 7	274.1	162.9 to 385.3	****
3 vs. 8	308.6	192.5 to 424.8	****
4 vs. 5	105.3	8.105 to 202.5	*
4 vs. 6	140.4	35.41 to 245.3	**
4 vs. 7	267	162.0 to 372.0	****
4 vs. 8	301.5	191.3 to 411.7	****
5 vs. 6	35.09	−83.92 to 154.1	NS
5 vs. 7	161.7	42.69 to 280.7	**
5 vs. 8	196.2	72.57 to 319.9	****
6 vs. 7	126.6	1.157 to 252.1	*
6 vs. 8	161.1	31.27 to 291.0	**
7 vs. 8	34.52	−95.34 to 164.4	NS

CI, confidence interval; DoF, degrees of freedom. *, $P < 0.05$; **, $P < 0.01$; ***, $P < 0.001$; ****, $P < 0.0001$.

Table S3. Fig. 2 B

One-way ANOVA	
DoF	5,74
F	20.35
P	< 0.0001
Statistical significance	****

Holm–Sidak multiple comparison test	Mean difference	95% CI of difference	Summary
Uninjected vs. DMSO injected	−1.454	−137.0 to 134.1	NS
Uninjected vs. DMSO injected	−66.43	−168.5 to 35.61	NS
Uninjected vs. DMSO injected	20.74	−111.5 to 153.0	NS

CI, confidence interval; DoF, degrees of freedom. ****, P < 0.0001.

Table S4. Fig. 2 C

One-way ANOVA	
DoF	4,87
F	11.17
P	<0.0001
Statistical significance	****

Holm-Sidak multiple comparisons test (compared to DMSO injected)	Uninjected	10 μM HG bath	10 μM HG injected	100 μM HG injected
n	14	23	21	21
t	2.183	4.319	5.679	5.598
Statistical significance	*	****	****	****

CI, confidence interval; DoF, degrees of freedom; *n*, number of oocytes. *, P < 0.05; ****, P < 0.0001.

Table S5. Fig. 2 D

Ordinary two-way ANOVA			
Alpha	0.05		
**Source of variation**	**% of total variation**	**P value**	**P value summary**
Interaction	6.148	0.0141	*
Row factor	7.061	<0.0001	****
Column factor	12.82	<0.0001	****

Dunnett multiple comparison test	Mean difference	95% CI of difference	Summary
**Row 1**			
Uninjected vs. DMSO injected	20.74	−11.26 to 52.75	NS
Uninjected vs. 10 μM HG bath	26.73	−2.670 to 56.13	NS
Uninjected vs. 10 μM HG injected	19.4	−9.642 to 48.45	NS
Uninjected vs. 100 μM HG injected	30.16	−1.169 to 61.50	NS

**Row 2**			
Uninjected vs. DMSO injected	16.16	−14.09 to 46.40	NS
Uninjected vs. 10 µM HG bath	3.837	−21.83 to 29.50	NS
Uninjected vs. 10 µM HG injected	16.16	−9.741 to 42.06	NS
Uninjected vs. 100 µM HG injected	33.6	7.705 to 59.50	**
**Row 3**			
**Uninjected vs. DMSO injected**	−11.75	−43.08 to 19.59	NS
Uninjected vs. 10 µM HG bath	13.88	−13.06 to 40.82	NS
Uninjected vs. 10 µM HG injected	31.76	4.592 to 58.92	*
Uninjected vs. 100 µM HG injected	35.41	8.246 to 62.57	**
**Row 4**			
Uninjected vs. DMSO injected	−20.06	−52.14 to 12.01	NS
Uninjected vs. 10 µM HG bath	18.67	−6.998 to 44.33	NS
Uninjected vs. 10 µM HG injected	33	7.102 to 58.90	**
Uninjected vs. 100 µM HG injected	32.19	6.286 to 58.09	**

CI, confidence interval. *, P < 0.05; **, P < 0.01.

Table S6. Fig. 3 A

**Unpaired two-tailed *t* test**	
P value	<0.0001
Summary	****
One- or two-tailed P value?	Two tailed
t, DoF	4.847, 34.94

DoF, degrees of freedom. ****, P < 0.0001.

Table S7. Fig. 3 B

**Unpaired two-tailed *t* test**	
P value	<0.0001
Summary	****
One- or two-tailed P value?	Two tailed
t, DoF	8.731, 24.89

DoF, degrees of freedom. ****, P < 0.0001.

Table S8. Fig. 3 E

**Ordinary one-way ANOVA**	
DoF	3,51
F	17.6
P	<0.0001
Statistical significance	****

**Bonferroni multiple comparison test**	**Uninjected vs. 100 µM HG 9-91-01**	**Sh WF M356C vs. Sh WF M356C + 100 µM HG**
n1, n2	16, 21	8, 10
P	<0.0001	<0.0001
Statistical significance	****	****

DoF, degrees of freedom; n, number of oocytes. ****, P < 0.0001.

Table S9. Fig. 3 F

**Unpaired two-tailed *t* test**	
P value	0.5414
Summary	NS
One- or two-tailed P value?	Two tailed
t, DoF	0.6395. 7.561

DoF, degrees of freedom.

Table S10. Fig. 3 G

**Unpaired two-tailed *t* test**	
P value	0.0324
Summary	*
One- or two-tailed P value?	Two tailed
t, DoF	3.637, 3.183

DoF, degrees of freedom. *, P < 0.05.

Table S11. Fig. 3 H

**Unpaired two-tailed *t* test**	
P value	0.0085
Summary	**
One- or two-tailed P value?	Two tailed
t, DoF	3.318, 9.379

DoF, degrees of freedom. **, P < 0.01.

Table S12. Fig. 3 I

**Unpaired two-tailed *t* test**	
P value	0.0011
Summary	**
One- or two-tailed P value?	Two tailed
t, DoF	4.830, 8.400

DoF, degrees of freedom. **, P < 0.01.

Table S13. Fig. 5 B

**Ordinary two-way ANOVA**			
Alpha	0.05		
**Source of variation**	**% of total variation**	**P value**	**P value summary**
Interaction	3.398	0.0171	*
Row factor	3.763	0.0009	***
Column factor	82.38	<0.0001	****

Tukey multiple comparison test	Mean difference	95% CI of difference	Summary
**1**			
Vegetal vs. animal	132.2	94.55 to 169.9	****
Vegetal vs. melanin	133.1	95.45 to 170.8	****
Animal vs. melanin	0.9	−36.78 to 38.58	NS
**2**			
Vegetal vs. animal	98.44	67.67 to 129.2	****
Vegetal vs. melanin	143.7	112.9 to 174.5	****
Animal vs. melanin	45.25	14.49 to 76.02	**
**3**			
Vegetal vs. animal	98.42	74.59 to 122.2	****
Vegetal vs. melanin	105.7	70.63 to 140.8	****
Animal vs. melanin	7.291	−27.79 to 42.37	NS
**4**			
Vegetal vs. animal	114.6	76.88 to 152.2	****
Vegetal vs. melanin	171.7	134.0 to 209.4	****
Animal vs. melanin	57.15	19.47 to 94.83	**

CI, confidence interval. *, P < 0.05; **, P < 0.01; ***, P < 0.001; ****, P < 0.0001.

Table S14. Fig. 5 C

**Ordinary one-way ANOVA**	
DoF	(2,9)
F	74.64
P	<0.0001
Statistical significance	****

Tukey multiple comparisons test	Mean difference	95% CI of difference	Summary
Vegetal vs. animal	132.2	97.21 to 167.2	****
Vegetal vs. melanin	133.1	98.11 to 168.1	****
Animal vs. melanin	0.9	−34.11 to 35.91	NS

CI, confidence interval; DoF, degrees of freedom. ****, P < 0.0001.

Table S15. Fig. 5 D

**Ordinary one-way ANOVA**	
DoF	(2,9)
F	47.55
P	<0.0001
Statistical significance	****

Tukey multiple comparison test	Mean difference	95% CI of difference	Summary
Vegetal vs. animal	114.6	64.48 to 164.6	****
Vegetal vs. melanin	171.7	121.6 to 221.8	****
Animal vs. melanin	57.15	7.081 to 107.2	*

CI, confidence interval; DoF, degrees of freedom. *, P < 0.05; ****, P < 0.0001.

Table S16. Fig. 6 C

**Unpaired two-tailed *t* test**	
P	0.7365
Summary	NS
Significantly different? ($P < 0.05$)	No
One- or two-tailed P value?	Two tailed
t, DoF	0.3444, 12

DoF, degrees of freedom.

Table S17. Fig. 6 D

**Ordinary one-way ANOVA**	
DoF	(2,11)
F	1.718
P	0.2243
Statistical significance	NS

**Tukey multiple comparison test**	**Mean difference**	**95% CI of difference**	**Significant?**	**Summary**
Vegetal vs. animal	109.5	−353.9 to 572.9	No	NS
Vegetal vs. melanin	296.9	−140.0 to 733.8	No	NS
Animal vs. melanin	187.4	−276.0 to 650.9	No	NS

CI, confidence interval; DoF, degrees of freedom.

Table S18. Fig. 6 E

**Ordinary one-way ANOVA**	
DoF	(2,11)
F	1.414
P	0.2879
Statistical significance	NS

**Tukey multiple comparison test**	**Mean difference**	**95% CI of difference**	**Significant?**	**Summary**
Vegetal vs. animal	−12.56	−56.63 to 31.51	No	NS
Vegetal vs. melanin	−25.58	−67.39 to 16.23	No	NS
Animal vs. melanin	−13.02	−54.83 to 28.79	No	NS

CI, confidence interval; DoF, degrees of freedom.

Table S19. Fig. 6 F

**Ordinary one-way ANOVA**	
DoF	(2,11)
F	55.36
P	<0.0001
Statistical significance	****

**Tukey multiple comparison test**	**Mean difference**	**95% CI of difference**	**Significant?**	**Summary**
Vegetal vs. animal	−2.732	−8.677 to 3.213	No	NS
Vegetal vs. melanin	17.91	12.30 to 23.51	Yes	****
Animal vs. melanin	20.64	14.70 to 26.59	Yes	****

CI, confidence interval; DoF, degrees of freedom. ****, $P < 0.0001$.
